# Indocyanine Green Fluorescence Imaging in Prevention of Colorectal Anastomotic Leakage: A Randomized Clinical Trial

**DOI:** 10.1001/jamasurg.2025.0006

**Published:** 2025-03-05

**Authors:** Juha K. A. Rinne, Heikki Huhta, Tarja Pinta, Arto Turunen, Anne Mattila, Kyösti Tahkola, Olli Helminen, Pasi Ohtonen, Tero Rautio, Jyrki Kössi

**Affiliations:** 1 Department of Surgery, Päijät-Häme Central Hospital, Lahti, Finland; 2 University of Tampere, Tampere, Finland; 3 Department of Surgery, Oulu University Hospital and University of Oulu, Oulu, Finland; 4 Department of Surgery, Seinäjoki Central Hospital, Seinäjoki, Finland; 5 Department of Surgery, Kanta-Häme Central Hospital, Hämeenlinna, Hämeenlinna, Finland; 6 Department of Surgery, Central Hospital of Central Finland, Jyväskylä, Finland; 7 Research Service Unit, Oulu University Hospital, Oulu, Finland; 8 Translational Medicine Research Unit, University of Oulu, Oulu, Finland; 9 Department of Surgery, Oulu University Hospital, Oulu, Finland; 10 Translational Medicine Research Unit, Medical Research Center Oulu, University of Oulu, Oulu, Finland

## Abstract

**Importance:**

Performing a bowel anastomosis is a critical step in colorectal resection. Assessing the risk of anastomotic leakage remains challenging, even for experienced surgeons.

**Objective:**

To evaluate the use of indocyanine green (ICG) fluorescence imaging in assessing perfusion at the anastomotic site before and after anastomosis and determine whether it helps reduce anastomotic leakages.

**Design, Setting, and Participants:**

ICG-COLORAL is a prospective, randomized, multicenter study conducted from September 1, 2018, to December 31, 2023. Participants were recruited during preoperative outpatient clinic visits by clinicians not involved in the study, as well as by researchers. Participants were elective patients scheduled for laparoscopic resections, excluding low anterior resections, with planned primary anastomosis in 5 Finnish public hospitals experienced in laparoscopic colorectal surgery.

**Intervention:**

The intervention group received 5 mg of ICG intravenously before and after anastomosis formation. The fluorescence signal was assessed with a near-infrared–capable camera. The control group did not receive ICG fluorescence imaging.

**Main Outcome and Measure:**

The primary outcome measure was the anastomotic leak rate as detected by computed tomography.

**Results:**

Among 1136 patients in the intention-to-treat population, 526 (46.3%) were female and 610 (53.7%) male; they had a mean (SD) age of 70 (11) years, body mass index of 28 (5), and age-adjusted Charlson Comorbidity Index of 5 (3). Overall, the anastomotic leak rate was 5.8% (33/567) in the ICG fluorescence imaging group vs 7.9% (45/569) in the control group (odds ratio [OR], 0.73; 95% CI, 0.48-1.13; *P* = .16). For right-sided operations, the anastomotic leak rate with ICG fluorescence imaging was 5.9% (16/273) vs 6.7% (20/298) in the control group (OR, 0.87; 95% CI, 0.46-1.65). For left-sided operations, the anastomotic leak rate was 5.2% (14/267) with ICG fluorescence imaging vs 9.5% (23/243) without (OR, 0.55; 95% CI, 0.29-1.05). No patients reported adverse events related to ICG.

**Conclusions and Relevance:**

This study found that routine use of ICG fluorescence imaging does not significantly reduce the overall anastomotic leak rate in laparoscopic colorectal surgery if low anterior resections are excluded but may be beneficial in left-sided operations.

**Trial Registration:**

ClinicalTrials.gov Identifier: NCT03602677

## Key Points

**Question:**

Does the routine use of indocyanine green (ICG) fluorescence imaging decrease the rate of anastomotic leakages in laparoscopic colorectal surgery if low anterior resections are excluded?

**Findings:**

In this randomized clinical trial involving 1136 patients, using ICG fluorescence imaging did not reduce anastomotic leakages compared with clinical evaluation alone.

**Meaning:**

Routine use of ICG fluorescence imaging does not decrease the overall rate of anastomotic leak in laparoscopic colorectal surgery if low anterior resections are not included.

## Teaser

This randomized clinical trial evaluates the use of indocyanine green fluorescence imaging in assessing perfusion at the anastomotic site to determine whether it helps reduce anastomotic leakages in laparoscopic colorectal surgery if low anterior resections are excluded.

## Introduction

Anastomotic leakage is a severe complication of colorectal surgery, occurring in 2.8% to 19% of patients.^1^ It not only serves as a critical factor in determining a patient’s prognosis, but also significantly impacts their overall quality of life.^2,3^ Anastomotic leakage triples the financial cost of the operation.^4,5^ Moreover, it profoundly affects oncological outcomes, potentially delaying or preventing the timely administration of adjuvant chemotherapy.^6,7^

Clinical assessment of anastomotic leak risk is challenging even for experienced surgeons.^8^ Indocyanine green (ICG) is a water-soluble fluorescent dye that binds to plasma proteins and is an excellent tool for assessing tissue perfusion. ICG absorbs and emits near-infrared light that can be visualized by special near-infrared fluorescence imaging devices such as modern laparoscopic cameras. Traditionally, surgeons assess anastomosis viability based on bowel color and arterial bleeding from the transected end of the bowel. ICG fluorescence imaging has been used to enhance perfusion assessment at planned anastomotic sites for more than a decade.

Most studies on ICG fluorescence imaging are retrospective, focused on left-sided operations and rectal resections, and include only a small number of patients. Meta-analyses and systematic reviews suggest a beneficial effect of ICG fluorescence imaging on reducing anastomotic leakage and complications. Intraoperative ICG imaging appears to halve anastomotic leak risk, with reported rates around 3.8% with ICG fluorescence imaging vs 7.5% in colorectal surgery^9^ and 9% vs 13.9% in rectal resections, respectively.^10^ The results of randomized clinical trials (RCTs) are conflicting and mainly derived from rectal resections.^11,12,13^ There is only 1 RCT also including right-sided colectomies.^14^

The primary aim of our study was to compare the anastomosis leak rate in colorectal surgery while excluding low anterior resections. Anastomosis perfusion was assessed by ICG fluorescence imaging compared with clinical assessment alone. Secondary aims involved assessing variables such as the severity of anastomosis leakage, hospital readmission rate, reoperation rate, complications graded by Clavien-Dindo classification, and operation time. Our hypothesis was that the use of ICG fluorescence imaging decreases the anastomotic leakage rate.

## Methods

### Study Design

The ICG-COLORAL study was a prospective, randomized, multicenter study conducted at 4 Finnish public-funded secondary care hospitals, Päijät-Häme Central Hospital, Seinäjoki Central Hospital, Central Finland Central Hospital, and Kanta-Häme Central Hospital, and in 1 tertiary care hospital, Oulu University Hospital. All participating hospitals are administered by Finnish Well-being Service Counties, and the study did not receive monetary reimbursement from any commercial entity. The study was designed according to the Declaration of Helsinki and approved by the ethics committee of Oulu University. Because of the rarity of adverse events related to the use of ICG, a data monitoring committee was not appointed. The study was registered in Clinical Trials.gov (NCT03602677), and the trial protocol appears in Supplement 1.

Inclusion criteria for study recruitment were all elective patients with any colorectal pathology scheduled for elective laparoscopic colorectal resection with primary anastomosis. Exclusion criteria were planned anastomosis below the peritoneal fold, inflammatory bowel disease, planned end ostomy, planned diverting stoma, planned laparotomy, active abscess or enteric fistula, and previous serious adverse events related to intravenous iodine use.

The patients received written information concerning the study, were given the opportunity to ask questions about the study, and gave their written consent. Patient gender was determined by their Finnish social security number identifying them as either male or female.

### Randomization and Masking

Patients were randomly allocated (1:1 ratio) to study groups using sealed, consecutively numbered opaque envelopes sent to each study site by a research nurse. A biostatistician not involved in the clinical care of the patients prepared the randomization list using a computerized random number generator. Allocation was stratified by site and blocked within strata using random permuted blocks (block size 4, 6, 8, and 10). Enrollment was conducted during preoperative outpatient clinic visits by clinicians not involved in the study as well as by researchers. Consequently, information regarding refusals to participate and the number of excluded patients was not recorded in a reliable way. After informed consent was obtained, the envelope was opened, and the patient was randomized either to receive ICG fluorescence imaging during the operation or to serve as a control. The study design is shown in the Figure. The surgeon performing the operation was determined based on routine daily clinical practice independently from the study. The principal investigator at each center learned the overall group allocation only after the operation.

**Figure.**
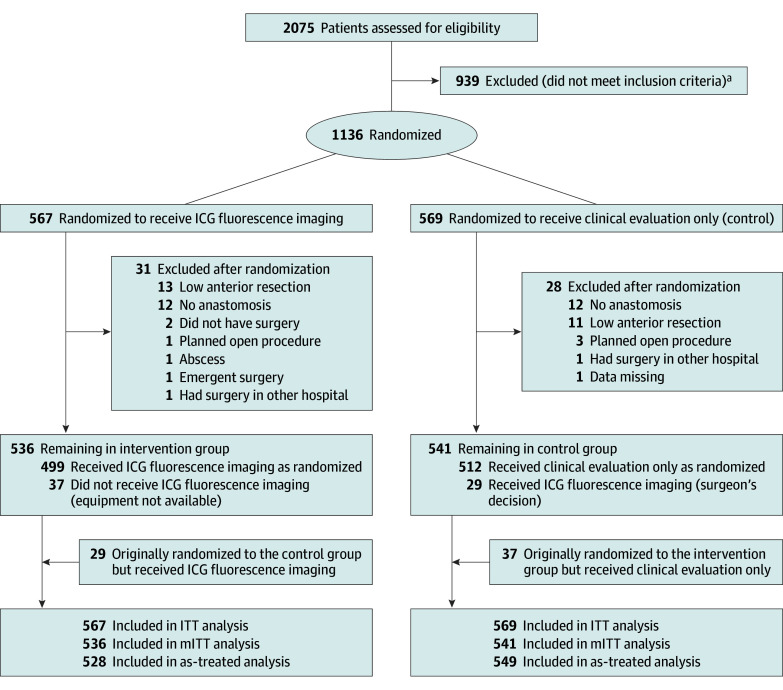
CONSORT Flowchart ICG indicates indocyanine green; ITT, intention-to-treat; mITT, modified intention-to-treat. ^a^Reasons for exclusion included planned open procedure, planned stoma, active abscess, enteric fistula, and anastomosis below peritoneal fold. Precise numbers per reason are not available because of the study design, conducted at 5 hospitals with more than 20 surgeons over 5 years. ICG indicates indocyanine green; ITT, intention-to-treat; mITT, modified intention-to-treat.

### Procedure

Oncologic resection followed the principles of radical surgery, including central vessel ligation and dissection along the embryologic planes. Left-sided resections with anastomosis to the upper third of the rectum were carried out in the total mesorectal excision plane. Resections of the middle or lower rectum were not included in the study. For procedures involving benign lesions, a similar approach was used, with the level of vessel ligation left to the discretion of the operating surgeon.

All patients received intravenous antibiotic prophylaxis; peroral antibiotics were not used. Päijät-Häme Central hospital used mechanical bowel preparation for all elective patients. No other hospital used bowel preparation for right-sided operations. Seinäjoki Central Hospital used an enema for left-sided colectomies while all the other hospitals used mechanical bowel preparation for left-sided operations.

#### Treatment Arm

A dose of 5 mg of ICG (Verdye; Diagnostic Green) was administered intravenously, and the seconds to maximal perceived visual signal intensity was recorded.

In the subjective ICG intensity scale (0-2), zero indicates no detected fluorescence, while 2 indicates fluorescence similar to that observed in the nonoperated bowel area. An intensity score of 1 represents an intermediate level, similar to the protocol by De Nardi et al.^11^

Both the time to maximal intensity and the intensity score at the clinically planned anastomotic site before bowel transection were recoded. The need to change the anastomotic site for any reason was also noted.

According to the study protocol, a revision was performed if the ICG fluorescence intensity score was 0, indicating no perfusion. For an intensity score of 1, revision was at the surgeon’s discretion, as factors such as the distance of the camera from the anastomosis could affect the intensity. After anastomosis completion, the administration of ICG was repeated. The estimated intensity score and time to maximal intensity in seconds were recorded for both the proximal and distal bowel segments of the anastomosis.

The cameras with near-infrared detection capability used in this study were the Stryker 1688 4K camera with advanced imaging modality and Pinpoint camera (Stryker), EinsteinVision 3.0 fluorescence imaging (Aesculap), Olympus Endoeye 10 mm, Endoeye 10 mm Exera III, Medtronic EleVision IR Platform, and DaVinci Xi.

#### Control Arm

No additional steps were required in assessing perfusion aside from not using ICG fluorescence imaging. Surgeons assessed perfusion based on their standard practices, such as evaluating the color of the bowel and checking for bleeding from the transected end. Revision of the anastomosis for any reason was noted.

### Outcomes

The primary outcome was the rate of anastomotic leakage. All the information was sent to Päijät-Häme Central Hospital and was subsequently analyzed by a statistician and an author (J.K.). Anastomotic leakage was diagnosed by triple-contrast computed tomography. Leakage severity was graded as local infection without abscess, abscess without clear intraluminal bowel connection, abscess with intraluminal bowel connection, or free perforation to abdominal cavity. Secondary outcomes were change of transection site of the bowel for any reason, time to maximal fluorescence signal, conversion rate, overall complications according to Clavien-Dindo classification, time to first flatus, bowel movement, length of postoperative hospital stay, 30-day readmissions, and 30-day mortality.

### Statistical Analysis

Power analysis for the study was based on anastomotic leak rates reported in a review by van den Bos et al.^15^ The anastomotic leakage rate was estimated to be 7.4% without ICG fluorescence imaging and 3.5% with ICG fluorescence imaging. Assuming 80% power and an α level of .05, 1062 patients were required for the study. Considering an estimated dropout rate of 10%, the projected need for enrollment was 1180 patients.

All analyses were primarily performed according to the intention-to-treat (ITT) principle. The ITT analysis compared the treatment and control arms as per the original randomization, with any potential crossovers analyzed according to their initially assigned groups. Randomization was conducted based on ICG allocation rather than by study center. Consequently, participants from all centers were analyzed collectively within their respective treatment or control arms.

Because of the recruitment method, some patients who should not have been recruited were also randomized. The most common reasons were no planned primary anastomosis or planned resection of the middle or lower part of the rectum. A modified ITT (mITT) analysis was performed, excluding these patients.

In accordance with the study protocol, an analysis based on the treatment received, referred to as the as-treated analysis, was also performed. In this analysis, patients were analyzed in the intervention group if they received ICG fluorescence imaging during the operation for any reason and in the control group if ICG fluorescence imaging was not performed.

An independent samples *t* test was used to compare means, and Pearson χ^2^ test was used to compare categorical variables. Additionally, a study-site–adjusted logistic regression analysis was performed because of the randomization protocol. Adjusted analysis was performed only for the primary outcome. Odds ratios (ORs) with 95% CIs are presented as a result for the logistic regression analysis. SPSS for Windows version 29 (IBM) was used for statistical analyses.

## Results

Patients were recruited between September 1, 2018, and December 31, 2023. A total of 2075 patients were operated on during this period, 939 of whom did not meet the inclusion criteria because of a planned open procedure, planned ostomy, active abscess, fistula, or resection of the middle or lower rectum. A total of 1136 patients were included in the ITT analysis. After the exclusion of 59 patients because of protocol violations, missing data, end ostomy (Hartman procedure), or rectal resection, 1077 patients were included in the mITT and as-treated analyses. There were no reported adverse events in patients given ICG intravenously.

ITT population baseline data, a side-by-side comparison of all groups, and the operative details of the ITT population are provided in eTables 1 and 2 in Supplement 2. The baseline characteristics of the mITT and as-treated populations are shown in Table 1. Among 1136 patients in the intention-to-treat population, 526 (46.3%) were female and 610 (53.7%) male; they had a mean (SD) age of 70 (11) years, body mass index of 28 (5), and age-adjusted Charlson Comorbidity Index of 5 (3). Most patients had American Society of Anesthesiologists classifications 2 and 3. The proportion of current smokers and the prevalence of benign and malignant tumors were similar, with no difference in the American Joint Committee on Cancer tumor stage. The mITT and as-treated groups’ intraoperative parameters are detailed in Table 2 and Table 3. The majority of right-sided anastomoses were performed in an isoperistaltic side-to-side fashion with a linear stapler, either openly through the specimen extraction site or laparoscopically with an endostapler. Four of 5 left-sided anastomoses were performed using a circular stapler, while the remainder were completed using a linear stapler or hand suturing (eTable 3 in Supplement 2).

**Table 1.** Baseline Characteristics in the mITT vs As-Treated Populations

Characteristic	mITT, No./total No. (%)		As-treated, No./total No. (%)	
	ICG fluorescence imaging group	Control	ICG fluorescence imaging group	Control
Gender				
Male	297 (55.4)	284 (52.5)	289 (54.7)	292 (53.2)
Female	239 (44.6)	257 (47.5)	239 (45.3)	257 (46.8)
BMI, mean (SD)^a^	27.6 (5)	27.6 (5)	27.7 (5)	27.6 (5)
Age, mean (SD), y	70 (11)	70 (11)	70 (11)	70 (11)
Age-adjusted CCI, mean (SD)	5 (2)	5 (3)	5 (2)	5 (3)
ASA class				
1	26 (4.9)	33 (6.1)	27 (5.1)	32 (5.9)
2	249 (46.6)	224 (41.5)	241 (45.7)	232 (42.4)
3	237 (44.4)	255 (47.2)	237 (45.0)	255 (46.6)
4	22 (4.1)	28 (5.2)	22 (4.2)	28 (5.1)
Smoker	55 (10.4)	63 (11.7)	57 (10.9)	61 (11.2)
Pathology				
Malignant	380 (70.9)	383 (70.8)	371 (70.3)	395 (71.9)
Benign	156 (29.1)	158 (29.2)	157 (29.7)	154 (28.1)
Tumor stage (AJCC)				
1	70 (19.7)	82 (22.3)	72 (20.9)	80 (21.2)
2	140 (39.4)	132 (36.0)	131 (38.0)	141(37.4)
3	117 (33.0)	130 (35.4)	115 (33.3)	132 (35.0)
4	28 (7.9)	23 (6.3)	27 (7.8)	24 (6.4)
Surgeon experience				
<10 y	287/534 (53.8)	290/540 (53.7)	285/527 (54.1)	287/547 (52.5)
>10 y	247/534 (46.3)	250/540 (46.3)	242/527 (45.9)	260/547 (47.5)
Previous laparoscopic colectomies				
No. of cases <50	171 (32.0)	166 (30.8)	162/527 (30.7)	173/546 (31.7)
No. of cases >50	361 (67.6)	370 (68.6)	365/527 (69.3)	373/546 (68.3)

Abbreviations: AJCC, American Joint Committee on Cancer; ASA, American Society of Anesthesiologists class; BMI, body mass index; CCI, Charlson Comorbidity Index; ICG, indocyanine green; mITT, modified intention-to-treat.

^a^ Calculated as weight in kilograms divided by height in meters squared.

**Table 2.** Operative Details for the mITT Population

Parameter	No./total No. (%)		*P* value
	ICG fluorescence imaging group	Control group	
Intraoperative complication	23/525 (4.4)	30/530 (5.7)	.34
Blood loss, mean (SD), mL	82 (110)	84 (146)	.73
Change of anastomotic site	38/527 (7.2)	23/527 (4.4)	.048
Conversions	30/536 (5.6)	23/541 (4.3)	.33
Protective stoma	4/531 (0.8)	10/536 (1.9)	.11
Operation			
Right-sided	274/536 (51.5)	294/541 (54.3)	.16
Left-sided	250/536 (46.6)	232/541 (42.9)	
Other (colectomies, transversum resections)	12/536 (2.3)	15/541 (2.8)	
Operation time, mean (SD), min	160 (60)	155 (63)	.18
Overall anastomotic leakage	33/536 (6.2)	45/541 (8.3)	.17
Right-sided	16/274 (5.8)	20/294 (6.8)	
Left-sided	14/250 (5.6)	23/232 (9.9)	
Postoperative infectious complications			
Antibiotics only	2/381 (0.5)	3/389 (0.8)	.68
Percutaneous drainage	3/381 (0.8)	3/389 (0.8)	
Reoperation	31/381 (8.1)	41/389 (10.5)	
Overall reoperations	52/536 (9.7)	54/540 (10.0)	.87
Overall complications	178/533 (33.4)	195/535 (36.4)	.30
Clavien-Dindo classification			
I	30/437 (6.9)	43/452 (9.5)	.56
II	113/437 (25.9)	110/452 (24.3)	
III	54/437 (12.4)	58/452 (12.8)	
IV	7/437 (1.6)	6/452 (1.3)	
V	3/437 (0.7)	7/452 (1.5)	
Postoperative hospital stay, mean (SD), d	5.6 (5)	5.6 (5)	.93
Anastomoses			
Intracorporeal, side-to-side, endostapler	160/536 (29.9)	175/541 (32.2)	
Extracorporeal, side-to-side, linear stapler	132/536 (24.7)	140/541 (25.9)	
Circular stapler	215/536 (40.1)	192/541 (35.5)	
Hand-sewn anastomosis	29/536 (5.4)	34/541 (6.3)	

Abbreviations: ICG, indocyanine green; mITT, modified intention-to-treat.

**Table 3.** Operative Details for the As-Treated Population

Parameter	No./total No. (%)		*P* value
	ICG fluorescence imaging group	Control group	
Intraoperative complication	21/519 (4.0)	32/536 (6.0)	.15
Blood loss, mean (SD), mL	78 (108)	88 (147)	.25
Change of anastomotic site	38/525 (7.2)	23/529 (4.3)	.045
Conversions	27/528 (5.1)	23/549 (4.2)	.48
Protective stoma	5/526 (1.0)	9/541 (1.7)	.31
Operation			
Right-sided	271/528 (51.3)	297/549 (54.1)	.20
Left-sided	245/528 (46.4)	237/549 (43.2)	
Other (colectomies, transversum resections)	12/528 (2.3)	15/549 (2.7)	
Operation time, mean (SD), min	158 (58)	158 (65)	.94
Overall anastomotic leakage	31/528 (5.9)	47/549 (8.6)	.09
Right-sided	15/271 (5.5)	21/297 (7.1)	
Left-sided	13/245 (5.3)	24/237 (10.1)	
Postoperative infectious complications			
Antibiotics only	3/528 (0.6)	2/549 (0.4)	.34
Percutaneous drainage	3/528 (0.6)	3/549 (0.5)	
Reoperation	28/528 (5.3)	44/549 (8.0)	
Overall reoperations	49/528 (9.3)	57/548 (10.4)	.54
Overall complications)	183/525 (34.9)	208/544 (38.2)	.25
Clavien-Dindo classification			
I	26/528 (4.9)	35/549 (6.4)	.67
II	113/528 (21.4)	110/549 (20.0)	
III	52/528 (9.8)	60/549 (10.9)	
IV	6/528 (1.1)	7/549 (1.3)	
V	3/528 (0.6)	7/549 (1.3)	
Postoperative hospital stay, mean (SD), d	5.6 (5)	5.6 (5)	>.99
Anastomoses			
Intracorporeal, side-to-side, endostapler	152/528 (28.8)	183/549 (33.3)	
Extracorporeal, side-to-side, linear stapler	131/528 (24.8)	141/549 (25.6)	
Circular stapler	217/528 (41.1)	190/549 (34.6)	
Hand-sewn anastomosis	28/528 (5.3)	35/549 (6.4)	

Abbreviation: ICG, indocyanine green.

The ITT analysis, consisting of all the randomized patients, showed no statistical difference between the intervention and control groups in overall anastomotic leak rates (5.8% [33/567] vs 7.9% [45/569], respectively; difference, −2.1 percentage points; 95% CI, −5.1 to 0.9; *P* = .16). The study-site–adjusted OR was 0.72 (95% CI, 0.45 to 1.14; *P* = .16). For right-sided operations, the anastomotic leak rate with ICG fluorescence imaging was 5.9% (16/273) vs 6.7% (20/298) in the control group (difference, −0.9 percentage points; 95% CI, −4.9 to 3.3). For left-sided operations, the anastomotic leak rate was 5.2% (14/267) with ICG fluorescence imaging vs 9.5% (23/243) without (difference, −4.2 percentage points; 95% CI, −9.0 to 0.3). Additional measures in the ITT analysis are shown in eTables 4 and 5 in Supplement 2.

The mITT analysis excluded patients who met the study exclusion criteria. Otherwise, the analysis was carried out according to ITT principles. The overall leakages were 6.2% (33/536) with ICG fluorescence imaging vs 8.3% (45/541) without (difference, −2.2 percentage points; 95% CI, −5.3 to 1.0; *P* = .17). The study-site–adjusted OR was 0.72 (95% CI, 0.45 to 1.15; *P* = .17). For the right side, the anastomotic leak rate was 5.8% (16/274) in the intervention group vs 6.8% (20/294) in the control group (difference, −1.0 percentage points; 95% CI, −5.1 to 3.2). For the left side, the anastomotic leak rate was 5.6% with ICG fluorescence imaging (14/250) vs 9.9% (23/232) in the control group (difference, −4.3 percentage points; 95% CI, −9.4 to 0.5). Additional measures in the mITT analysis are shown in eTables 6 and 7 in Supplement 2.

The as-treated analysis considered whether the patient received ICG fluorescence imaging during the operation, regardless of preoperative randomization. The overall anastomotic leak rate was 5.9% (31/528) in the ICG fluorescence group vs 8.6% (47/549) in the control group (difference, −2.7 percentage points; 95% CI, −5.8 to 0.4; *P* = .09). The study-site–adjusted OR was 0.67 (95% CI, 0.42 to 1.08; *P* = .10). For the right-sided operations, the anastomotic leak rate was 5.5% (15/271) in the ICG fluorescence group vs 7.1% (21/297) in the control group (difference, −1.5 percentage points; 95% CI, −5.6 to 2.6). For the left-sided operations, the anastomotic leak rate was 5.3% (13/245) in the ICG fluorescence group vs 10.1% (24/237) in the control group (difference, −4.8 percentage points; 95% CI, −9.8 to 0.0). Additional measures in the as-treated analysis are shown in eTables 8 and 9 in Supplement 2.

We analyzed the effects of types of bowel preparation and type of anastomosis and found no statistically significant benefit in any of the analyses. In addition, a comparison of anastomotic leakages is provided in eTable 10 in Supplement 2.

## Discussion

To our knowledge, this study is the first large-scale RCT reporting ICG fluorescence imaging use in colorectal surgery excluding low anterior resections. ITT analysis showed no statistical difference in overall anastomotic leakage between the ICG fluorescence imaging and control groups, and this result remained consistent across the mITT and as-treated analyses. A potential benefit was observed in the as-treated analysis for left-sided colectomies; however, since this finding is derived from a subgroup analysis, definitive conclusions cannot be made.

Comparing our results to previous studies is somewhat challenging because all prior RCTs focus solely on rectal surgery or include a significant number of low anterior resections. Because the available data support the use of ICG in rectal surgery, we chose to focus mainly on colon surgery. Determining the exact distance of an anastomosis from the anal verge beforehand can be clinically challenging, so we decided to use the peritoneal fold as the boundary, which consequently includes some resections of the upper rectum.

The only RCT with a patient selection similar to ours is an excellent study by Faber et al,^14^ involving 982 patients undergoing colorectal resection. However, their study included roughly 20% of patients undergoing low anterior resection. In their trial, the overall 90-day rate of clinically relevant anastomotic leakage was 7% in the ICG fluorescence imaging group vs 9% in the control group (*P* = .24). In a subgroup analysis focusing on left-sided operations, the anastomotic leakage rate was 8% with ICG fluorescence imaging compared with 13% in the control group, showing a statistically significant difference (*P* = .047). No benefit of ICG fluorescence imaging was observed in right-sided operations: 4% with ICG fluorescence imaging vs 4% in the control group (*P* = .96). Although we excluded low anterior resections, our results are remarkably similar.

Two recent large systematic reviews focusing on ICG fluorescence imaging^9,10^ suggest that its use decreases anastomotic leak rates. However, this effect appears to be predominantly derived from studies including low anterior resections. Furthermore, studies by Watanabe et al^13^ and Alekseev et al^12^ highlighted that the use of ICG fluorescence imaging reduces anastomotic leakage, particularly in low rectal resections. While our results indicate a potential benefit in left-sided resections, they cannot be directly compared with these findings. Nevertheless, they do support prior evidence regarding the utility of ICG fluorescence imaging in left-sided operations.

In contrast to our trial, studies by Alekseev et al^12^ and De Nardi et al^11^ failed to demonstrate a benefit of ICG fluorescence imaging use in left-sided colorectal procedures when low anterior resections were excluded from the analysis. Both studies included only a small number of patients: the study by Alekseev et al involved 377 patients and that by De Nardi et al, 240 patients. These studies may have suffered from insufficient statistical power to adequately evaluate outcomes in left-sided colorectal procedures. Our study included approximately 500 left-sided procedures, but the study was not designed to analyze left-sided operations independently from other procedures, limiting the strength of our results.

### Limitations

Our study has several limitations. First, the assessment of ICG fluorescence imaging signal intensity relied on the surgeon’s subjective judgment, introducing a risk of human error. Second, a learning curve exists in assessing the ICG fluorescence imaging signal, with some experts suggesting it takes more than 50 cases.^16^ The use of ICG fluorescence imaging was novel to all surgeons at the study’s onset. Third, the signal intensity also varies based on the distance of the camera from the tissue and even the camera angle,^17^ none of which was standardized in our study. In the future, this could be alleviated by introducing artificial intelligence and machine learning models for the assessment of the ICG fluorescence imaging signal, as described by Dalli et al.^18^

Fourth, we did not have a screening list during recruitment, so precise information on the excluded patients was lacking. Despite predefined and relatively straightforward inclusion criteria, 5% of recruited patients did not meet them. This reflects the reality of conducting clinical trials in everyday practice, where recruitment occurs alongside routine clinical work by practitioners not directly involved in the trial. The study included 5 participating hospitals and more than 20 surgeons over a 5-year period. The data collection was incomplete for some participants, which is a well-known problem in large-scale multicenter clinical trials.^19^ Using electronic case report forms with required completion fields could reduce missing data incidence. Additionally, reducing the amount of data collected and using data validation techniques are ways to minimize missing data. Sensitivity analysis and multiple imputation are other statistical methods that can reduce the bias introduced by missing values, specifically addressing the risks of selection bias and information bias.^19^ However, in our study, the missing data were missing at random, so it is unlikely that our missing values were significantly different from our observed values.

## Conclusions

This study found that routine use of ICG fluorescence imaging does not significantly reduce the overall anastomotic leak rate in laparoscopic colorectal surgery if low anterior resections are excluded. We conclude that routine use of ICG fluorescence imaging in laparoscopic colon surgery does not appear to be warranted. However, it may offer potential benefits for left-sided colorectal operations in preventing anastomotic leakage.

## Notes

Supplement 1. Trial protocol

Supplement 2. **eTable 1.** Baseline characteristics ITT vs mITT vs AT **eTable 2.** Operative details ITT **eTable 3.** Comparison of anastomoses **eTable 4.** ITT: Time to maximal signal intensity seconds, mean (SD) **eTable 5.** ITT: Intensity scores 0-2. No. (%) **eTable 6.** mITT: Time to maximal signal intensity, seconds, mean (SD) **eTable 7.** mITT: Intensity scores 0-2. No. (%) **eTable 8.** As Treated: Time to maximal signal intensity, seconds, mean (SD) **eTable 9.** As Treated: Intensity scores 0-2. No. (%) **eTable 10.** Comparison of Anastomotic leakages

Supplement 3. Data sharing statement
