# Supplementary material for: Indocyanine Green Fluorescence Imaging in Prevention of Colorectal Anastomotic Leakage: A Randomized Clinical Trial
Source: JAMA Surg. 2025 Mar 5;160(5):486–93. doi: 10.1001/jamasurg.2025.0006 (PMC11883591; doi:10.1001/jamasurg.2025.0006)
Supplement: Supplement 1. — Trial protocol [file jamasurg-e250006-s001.pdf]

Title:

Indocyanine green fluorescence imaging in prevention of anastomosis leakages after colorectal surgery

Background:

Laparoscopic approach is routine method for colorectal bowel resection nowadays. It reaches to equal or better results as open surgery in both benign and malign conditions (1, 2). Anastomotic leakage (AL) is one of the most devastating complications of colorectal surgery. AL can result in increased morbidity and mortality and can adversely affect the length of hospital stay, cost and cancer recurrence (3). The reported rates of AL after colorectal surgery range from 3-19% (4). Risk factors for AL are male sex, level of anastomosis, tobacco use, preoperative chemotherapy, preoperative radiation and the occurrence of intraoperative adverse events (5).

Adequate vascular perfusion is of paramount importance for successful anastomosis and avoidance of intestinal ischemia. Surgeons have several subjective clinical checks for vascular perfusion, including examination of the color of the bowel wall, visible peristalsis, palpable pulsation and bleeding from the marginal arteries and stapled seam (6). Objective and accurate measurements of the intestinal perfusion are desirable to reduce AL (7). Near-infrared (NIR) fluorescence technology with indocyanine green (ICG) has become the most promising method that allows for an accurate evaluation of intestinal perfusion intraoperatively (8, 9)

ICG is a water-soluble, tricarbocyanine compound dye that absorbs NIR light at 800–810 nm and emits it at 830 nm. Under NIR light intravenous ICG injection becomes fluorescent, providing a “real-time” confirmation of intestinal perfusion. Therefore, it helps in determining the point of resection after mesenteric division and demonstrates the presence of an ischemic perfusion before performing the anastomosis (10).

Aims:

Main aim: Compare the anastomosis leakage rates in colorectal surgery after using ICG or not.

Secondary aims: Severity of anastomosis leakage, hospital readmission rates, reoperation rates, Clavien-Dindo score, operation time, length of hospital stay, 30- and 90-day mortality rates, time to first bowel movement (days), time to first flatus (days).

Methods:

Study design

The aims above will be tested in randomized controlled trial performed in six Finnish hospitals (Oulu, Jyväskylä. Lahti, Tampere, Turku, Jorvi). Patients eligible for the study are those with a diagnosed colonic or rectal pathology needing surgery and who are undergoing colon surgery with primary anastomosis, or rectal surgery with primary anastomosis.

Patient with right side pathology will be operated without bowel emptying, patient with left-side pathology will have clyster and patient with rectal pathology will undergo bowel emptying. All patients receive cefuroxime (or clindamycin if contraindicated) and metronidazole at induction. No separate preoperative antibiotic is given.

Inclusion criteria

All consenting patients in the catchment area of above mentioned hospitals who undergo elective colorectal surgery with planned primary anastomosis are eligible for this study. Of rectal cancer patients only those with pathology in the proximal third will be included in the study (defined by area proximal from peritoneal fold).

Exclusion criteria

Emergent patients, patients with proven diverticular abscess and colonic fistulas are excluded. Also patients with planned open surgery are excluded.

Patient randomization

Patients diagnosed with colorectal pathology are randomized at the preoperative visit to either ICG+ or ICG- group. Patient information is given and written informed consent will be received from all participants before randomization. Number of patients refusing to take part in the study is also collected. Total number of patients

operated due to colorectal pathology in participating centers will be reported, and reason for exclusion from the study.

<u>*Data collection*</u>

Following baseline data will be collected from all patients during the hospital admission or before: sex, age, comorbidity, smoking (no, ex, current), received neoadjuvant chemo- or chemoradiotherapy, previous operations (abdomen), reason for operation (diagnosis), stage and date of cancer diagnosis.

Following data will be collected regarding the operation: Type of operation, use of stoma or not, type of anastomosis, length of surgery, amount of bleeding, blood transfusions, peroperative complications, surgeon experience (number of performed laparoscopic bowel resections), ICG-staining intensity in planned resection line (scale 0-2), ICG-staining intensity after primary anastomosis on both sides of the anastomosis (scale 0-2), time from ICG injection to maximal intensity (at the planned site and after completion of the anastomosis).

In ICG intensity scale zero means no detected fluorescence and two means similar fluorescence as observed in proximal non-operated bowel area. Intensity of one is between these two.

Photograph is taken at the site of planned anastomosis and after the completion of the anastomosis.

If after ICG-staining the location of the resection line is changed, or if a new anastomosis is performed this will be collected also.

If anastomosis shows zero intensity a new anastomosis will be performed. With medium intensity (1) the operating surgeon decides the need for reanastomosis according to clinical situation.

All patients are followed for anastomosis leakage rates, severity of the anastomosis leakages, other complications (Clavien-Dindo score), time to first bowel movement

and flatus, length of hospital stay, hospital readmission rates, reoperation rates, and 30- and 90-day mortality rates.

Full-time study coordinator will collaborate weekly with all participating centers, collecting needed information of all newly operated / discharged patients. Afore mentioned parameters will be collected at preoperative visit (Table 2), at the operation room by nurse (Table 3) and at the time of discharge (by doctor dictating the epicrisis) supplemented at 90-days after the operation (by study assistant), Table 4.

Power analysis:

Total number of elective colorectal operations in above mentioned six centers is approximately 1000 patients / year. Of these all are screened and recruited for the study. We believe that majority of patients are willing participate the study, but some dropouts (estimated as 10%) will exist because of technical difficulties with ICG equipment and early conversion to laparotomy. Therefore power calculations have been made with 1000 patients / year. According to previous reports, anastomosis leakage rate after colon surgery is approximately 6-8% and after rectal surgery 10%. With ICG-staining we hypothesize that most anastomosis leakages can be significantly reduced. In recent systematic review van den Bos et al. (Journal of laparoendoscopic & advanced surgical techniques) reported anastomosis leakage rates of 7.4% without NIR imaging and 3.5% with performed NIR imaging. With these numbers the sample size of 1062 patients is needed with power of 80% and alpha 0.05. Both groups will include 531 patients. The study will need approximately 1.5 years until all patients have been recruited.

Exposures

Group 1: Colorectal surgery with primary anastomosis without ICG-use (reference group)

Group 2: Colorectal surgery with primary anastomosis with ICG-use (test group)

Study outcomes

Primary outcome

- Anastomosis leakage rate between groups.

Secondary outcomes

- Severity of anastomosis leakage, timing of anastomosis leakage, deep surgical site infections, hospital readmission rates, reoperation rates, Clavien-Dindo score, operation time, length of hospital stay, 30- and 90-day mortality rates, time to first bowel movement (days), time to first flatus (days) and hospital costs.

Anastomosis leakage is diagnosed by triple contrast CT in case of suspected ananstomosis leakage. Additionally colonoscopy may be performed. Anastomosis severity is graded as follows: local infection without abscess, abscess without clear intraluminal bowel connection, abscess with intraluminal bowel connection, free perforation to abdominal cavity.

Possible confounders

Because of the study design (randomized controlled trial including all patients with planned laparoscopic colorectal surgery with primary anastomosis) confounding factors should be excluded.

The following baseline information will be reported (See Table 2):

(1) Age (at the time of operation);
(2) Sex: male – female;
(3) Charlson Comorbidity Index: 0, 1, ≥2
(4) Tumor stage according to AJCC 8th edition.

1) <u>Comorbidity</u>

Comorbidity was defined as any disease the patient had before operation (maximum 3 years), or at the time of the operation. These will be classified as 0, 1 and ≥2 according to the Charlson Comorbidity Index (based on presence or absence of the following comorbidities (Table 3).

Table 3: The diagnoses used to calculate the Charlson's comorbidity index.

**Disease**	**ICD-10 codes**
Myocardial infarction	**I21*, I22*, I23*,** I252
Congestive cardiac failure	I11, I13, I255, I42, I43, I50, I517
Peripheral vascular disease	I70–I73, I770, I771, K551, K558, K559, R02, Z958, Z959
Cerebrovascular disease	G45, G46, I60–I69
Dementia	A810, F00–F03, F051, G30, G31
Chronic pulmonary disease	I26, I27, J40–J45, **J46***, J47, J60–J67, J684, J701, J703
Rheumatological disease	M05, M06, M09, M120, M315, M32–M36
Liver disease	B18, I85, I864, I982, K70, K71, K721, K729, K76, R162, Z944
Diabetes mellitus	E10–E14
Hemiplegia or paraplegia	G114, G81–G83
Renal disease	I12, I13, N01, N03, N05, N07, N08, **N171*, N172*,** N18, **N19***, N25, Z49, Z940, Z992
Any malignancy	C00–C26, C30–C34, C37–C41, C43, C45–C58, C60–C76, C80–C85, C88, C90–C97
Metastatic solid tumour	C77–C79
AIDS/HIV infection	B20–B24

*Will be only taken into account for previous, not current, hospital admissions

Colorectal cancers are not counted as comorbidites. The prevalence of metastatic diseases among the study is assessed first (sensitivity analysis) and then the inclusion or exclusion of these diseases into the Charlson's score is determined.

Statistical analyses

Descriptive statistics of patient characteristics will be presented separately for the two groups (ICG or not).

The amount of primary and secondary outcomes will be tested by chi-square test, Students T-test or Mann-Whitney U test as appropriate.

Subgroup analysis

Subgroup analyses will be conducted for the primary outcome by

1) Separately patients with colon and rectal pathology.
2) Separately patients with preoperative chemo/radiation.
3) Separate analysis according to Stage (I-III vs IV)
4) Separate comparison of patients in which the location of anastomosis was changed due to ICG staining to patients with performed ICG staining but no change in the anastomosis location, and to patients in no-ICG group.
5) Separate comparison of patients with slow appearing ICG-staining versus fast staining (divided with median time).
6) Separate analysis of patients with low and high comorbidity (CCI $\geq$ 2)
7) Separately according to age ($\geq$70 years).
8) ICG staining intensity in smokers vs non-smokers and correlation with leakage rate.
9) Separately patients with right and left resection
10) Separate analysis according to diagnosis

<u>Handling of missing data:</u>

There will be number of missing data due to inadequate data collection in all study centers. This will be minimized with active weekly data collection by study assistant and the researchers. Missing data will be reported.

Collaborators:

First Author: Juha Rinne

Last Author: Jyrki Kössi

Other: Olli Helminen (KSKS and OYS), Heikki Huhta (OYS), Matti Kairaluoma (KSKS), Tero Rautio (OYS), Tom Scheinin (Jorvi), Jukka Karvonen (TYKS), xx (TAYS).

Statistician: Pasi Ohtonen (OYS).

Timeframe:

Study protocol	December 2017
Required permissions	March 2018
Patient recruiting starts	July 2018
Patient recruiting completed	December 2019
Merging of the data	March 2020
Manuscript writing	April 2020

Tables

Table 1. Clinical variables by surgical technique in n patients operated with colorectal cancer resection with and without ICG.

Variable	Anastomosis with ICG	Anastomosis without ICG
	Patients (%)<br>n=	Patients (%)<br>n=
Mean age (SD)		
Sex		
Male		
Female		
Charlson comorbidity index		
0		
1		
≥2		
BMI		
Anastomosis leakage(AL) (n)		
Severity of AL[1]		
No leak		
Grade A		
Grade B		
Grade C		
Need of blood pressure support		
Intraoperative blood transfusion (units)		
Postoperative blood transfusion (units)		
Intraoperative bleeding		
Length of surgery		
Hospital stay (days)		
ICU stay (days)		
Re-operation rate		
Re-admissions		
First bowel moment (day)		
First flatus (day)		
30d mortality		
90d mortality		
Clavien-Dindo score		
Grade I		
Grade II		
Grade IIIa		
Grade IIIb		
Grade IVa		
Grade IVb		
Grade V		
Primary ICG staining intensity at the planned site		
Primary ICG staining intensity after performed anastomosis		
Time from ICG injection to maximal intensity		
Changing of anastomosis location		

according to ICG staining		
Preoperative chemotherapy and/or radiation		
Type of surgery		
Left hemicolectomy		
Right hemicolectomy		
Sigmoid resection		
Proximal rectum resection		
Formation of stoma		
Tobacco use		
Surgeon experience		
Tumor stage		
Location of the tumor		
Circular, linear, hand sued, intra- or extracorporeal anastomosis		
Anastomosis type		

[1]Grade A = No change in patient management, Grade B = leakage requires active therapeutic intervention but is manageable without re-laparotomy, Grade C = leakage requires re-laparotomy

Table 2. Preoperatiivisella poliklinikkakäynnillä kerättävät tiedot

Nimi, hetu	
Ikä	
Sukupuoli	
Pituus	
Paino	
Perussairaudet	
Tupakointi	
Aiemmat vatsan alueen operaatiot	
Leikkauksen syy (diagnoosi)	
Tuumorin tarkka sijainti	
Mahdollisen syövän diagnoosipäivämäärä	
Mahdollisen syövän stage	
Mahdollinen preoperatiivinen kemoterapia	
Mahdollinen preoperatiivinen sädehoito	

Table 3. Leikkaussalissa kerättävät tiedot

Leikkauksen TMP-koodi	
Leikkauksen pituus (min)	
Leikkausvuodon määrä (ml)	
Siirrettyjen punasoluyksiköiden määrä	
Verenpainetuen tarve (kyllä/ei)	
Mahdolliset leikkauksen aikaiset komplikaatiot	
Kirurgin kokemus (alle/yli 10 vuotta)	
ICG-värin intensiteetti suunnitellussa resektiolinjassa (asteikko: 0, 1, 2)	
ICG-värin intensiteetti anastamoosin proksimaali puolella	
ICG-värin intensiteetti anastamoosin distaalipuolella	
ICG-injektiosta maksimi-intensiteettiin kuluva aika suunnitellussa kohdassa (s)	
ICG-injektiosta maksimi-intensiteettiin kuluva aika anastomoosin proksimaalipuolella	
ICG-injektiosta maksimi-intensiteettiin kuluva aika anastomoosin distaalipuolella (s)	
Otettiinko kuva (kyllä/ei)	
Vaihdettiinko anastamoosin paikkaa (kyllä/ei)	
Tehtiinkö suoja-avannetta (kyllä/ei)	
Toteutettiinko suunniteltu leikkaus (kyllä/ei)	
Vesi-ilmatestin tulos	
Tekniset vaikeudet anastomoosin teossa	

Table 3. Potilasta kotiuttaessa kerättävät tiedot

Anastamoosilekaasin vaikeusaste	
0	Ei lekaasia
A	Ei toimenpiteitä lääkitystä lukuun ottamatta
B	Pieni toimenpide, esim. stenttaus, dreneeraus, punktio
C	Uusintaleikkaus
Reoperaatiolöydös	
	Ei iskemiaa sauman alueella
	paikallinen iskemia
	laajempi suoli-iskemia/nekroosi
Sairaalahoidon kesto (päiviä)	
Tehohoitopäivät (päiviä)	
Uusintaoperaatioiden määrä (kpl)	
Leikkauksen jälkeinen suolistokaasujen läpitulo (päiviä)	
Leikkauksen jälkeinen suolen toimiminen (päiviä)	
Post operatiivinen komplikaatio	
Siirrettyjen punasoluyksiköiden määrä	
Hengitystuen tarve (muu, kuin lisähappi)	
Pneumonia (kyllä/ei)	
Hoitoa vaatinut rytmihäiriö / sydäninfarkti (kyllä/ei)	
Vti (kyllä/ei)	
SLT / keuhkoembolia / aivoinfarkti (kyllä/ei)	
Delirium (kyllä/ei)	
Haavainfektio, ei salirevision tarvetta	
Haavainfektio, vaatinut salirevision	
Toimenpiteen vaatinut abskessi (kyllä/ei)	
Abskessi, ei vaatinut toimenpidettä (kyllä/ei)	
Faskian aukeaminen (kyllä/ei)	
Tarvinnut sairaalahoitoa kotiutumisen jälkeen (30 pv sisään) (kyllä/ei)	
Tarvinnut sairaalahoitoa kotiutumisen jälkeen (90 pv sisään) (kyllä/ei)	
Kuollut 30 pv sisään leikkauksesta (kyllä/ei)	
Kuollut 90 pv sisään leikkauksesta (kyllä/ei)	

References:

1. COLOR: a randomized clinical trial comparing laparoscopic and open resection for colon cancer. Digestive surgery. 2000;17(6):617-22.
2. van der Pas MH, Haglind E, Cuesta MA, Furst A, Lacy AM, Hop WC, et al. Laparoscopic versus open surgery for rectal cancer (COLOR II): short-term outcomes of a randomised, phase 3 trial. The Lancet Oncology. 2013;14(3):210-8.
3. Mirnezami A, Mirnezami R, Chandrakumaran K, Sasapu K, Sagar P, Finan P. Increased local recurrence and reduced survival from colorectal cancer following anastomotic leak: systematic review and meta-analysis. Annals of surgery. 2011;253(5):890-9.
4. Kingham TP, Pachter HL. Colonic anastomotic leak: risk factors, diagnosis, and treatment. Journal of the American College of Surgeons. 2009;208(2):269-78.
5. Qu H, Liu Y, Bi DS. Clinical risk factors for anastomotic leakage after laparoscopic anterior resection for rectal cancer: a systematic review and meta-analysis. Surgical endoscopy. 2015;29(12):3608-17.
6. Karliczek A, Harlaar NJ, Zeebregts CJ, Wiggers T, Baas PC, van Dam GM. Surgeons lack predictive accuracy for anastomotic leakage in gastrointestinal surgery. International journal of colorectal disease. 2009;24(5):569-76.
7. Urbanavicius L, Pattyn P, de Putte DV, Venskutonis D. How to assess intestinal viability during surgery: A review of techniques. World journal of gastrointestinal surgery. 2011;3(5):59-69.
8. Protyniak B, Dinallo AM, Boyan WP, Jr., Dressner RM, Arvanitis ML. Intraoperative indocyanine green fluorescence angiography--an objective evaluation of anastomotic perfusion in colorectal surgery. The American surgeon. 2015;81(6):580-4.
9. Jafari MD, Lee KH, Halabi WJ, Mills SD, Carmichael JC, Stamos MJ, et al. The use of indocyanine green fluorescence to assess anastomotic perfusion during robotic assisted laparoscopic rectal surgery. Surgical endoscopy. 2013;27(8):3003-8.
10. Nachiappan S, Askari A, Currie A, Kennedy RH, Faiz O. Intraoperative assessment of colorectal anastomotic integrity: a systematic review. Surgical endoscopy. 2014;28(9):2513-30.
