# Supplementary material for: Indocyanine Green Fluorescence Imaging in Prevention of Colorectal Anastomotic Leakage: A Randomized Clinical Trial
Source: JAMA Surg. 2025 Mar 5;160(5):486–93. doi: 10.1001/jamasurg.2025.0006 (PMC11883591; doi:10.1001/jamasurg.2025.0006)
Supplement: Supplement 2. — eTable 1. Baseline characteristics ITT vs mITT vs AT eTable 2. Operative details ITT eTable 3. Comparison of anastomoses eTable 4. ITT: Time to maximal signal intensity seconds, mean (SD) eTable 5. ITT: Intensity scores 0-2. No. (%) eTable 6. mITT: Time to maximal signal intensity, seconds, mean (SD) eTable 7. mITT: Intensity scores 0-2. No. (%) eTable 8. As Treated: Time to maximal signal intensity, seconds, mean (SD) eTable 9. As Treated: Intensity scores 0-2. No. (%) eTable 10. Comparison of Anastomotic leakages [file jamasurg-e250006-s002.pdf]

Supplemental Online Content

Rinne JKA, Huhta H, Pinta T, et al. Indocyanine green fluorescence imaging in prevention of colorectal anastomotic leakage: a randomized clinical trial. *JAMA Surg*. Published online March 5, 2025. doi:10.1001/jamasurg.2025.0006

eTable 1. Baseline characteristics ITT vs mITT vs AT

eTable 2. Operative details ITT

eTable 3. Comparison of anastomoses

eTable 4. ITT: Time to maximal signal intensity seconds, mean (SD)

eTable 5. ITT: Intensity scores 0-2. No. (%)

eTable 6. mITT: Time to maximal signal intensity, seconds, mean (SD)

eTable 7. mITT: Intensity scores 0-2. No. (%)

eTable 8. As Treated: Time to maximal signal intensity, seconds, mean (SD)

eTable 9. As Treated: Intensity scores 0-2. No. (%)

eTable 10. Comparison of Anastomotic leakages

This supplemental material has been provided by the authors to give readers additional information about their work.

© 2025 Rinne JKA et al. *JAMA Surgery*.

eTable 1. Baseline characteristics ITT vs mITT vs AT

	ICG FI ITT (n=568)	Control ITT (n=568)	ICG FI mITT (n=536)	Control mITT (n=541)	As Treated (n=528)	Control As Treated (n=549)
**Gender**						
**Male No. (%)**	314 (55.3)	296 (52.1)	297 (55.4)	284 (52.5)	289 (54.7)	292 (53.2)
**Female No. (%)**	254 (44.7)	272 (47.9)	239 (44.6)	257 (47.5)	239 (45.3)	257 (46.8)
**BMI (kg/m², mean, SD)**	27.7 (5)	27.6 (5)	27.6 (5)	27.6 (5)	27.7 (5)	27.6 (5)
**Age, years, mean (SD)**	70 (11)	70 (11)	70 (11)	70 (11)	70 (11)	70 (11)
**Age-adjusted CCI, mean (SD)**	5 (2)	5 (3)	5 (2)	5 (3)	5 (2)	5 (3)
**ASA No. (%)**						
**1**	27 (4.8)	33 (5.9)	26 (4.9)	33 (6.1)	27 (5.1)	32 (5.9)
**2**	263 (47.1)	235 (41.7)	249 (46.6)	224 (41.5)	241 (45.7)	232 (42.4)
**3**	245 (43.9)	268 (47.5)	237 (44.4)	255 (47.2)	237 (45.0)	255 (46.6)
**4**	23 (4.1)	28 (5.0)	22 (4.1)	28 (5.2)	22 (4.2)	28 (5.1)
**Active Smoker No. (%)**	59 (10.6)	64 (11.4)	55 (10.4)	63 (11.7)	57 (10.9)	61 (11.2)
**Pathology No. (%)**						
**Malignant**	395 (69.5)	401 (70.5)	380 (70.9)	383 (70.8)	371 (70.3)	395 (71.9)
**Benign**	173 (30.4)	168 (29.5)	156 (29.1)	158 (29.2)	157 (29.7)	154 (28.1)
**Tumor stage (AJCC) No. (%)**						
**1**	77 (20.9)	88 (23.0)	70 (19.7)	82 (22.3)	72 (20.9)	80 (21.2)
**2**	144 (39.0)	134 (35.1)	140 (39.4)	132 (36.0)	131 (38.0)	141(37.4)
**3**	120 (32.5)	137 (35.9)	117 (33.0)	130 (35.4)	115 (33.3)	132 (35.0)
**4**	28 (7.6)	23 (6.0)	28 (7.9)	23 (6.3)	27 (7.8)	24 (6.4)

© 2025 Rinne JKA et al. *JAMA Surgery*.

**Surgeon's Experience (years) No. (%)**						
**< 10 years**	299/554 (53.9)	300/557 (53.8)	287/534 (53.8)	290/540 (53.7)	285/527 (54.1)	287/547 (52.5)
**> 10**	255/554 (46.0)	257/557 (46.1)	247/534 (46.3)	250/540 (46.3)	242/527 (45.9)	260/547 (47.5)
**Previous lap.colectomies No (%)**						
**< 50 cases**	183/554 (33.1)	177/556 (31.8)	171 (32.0)	166 (30.8)	162/527 (30.7)	173/546 (31.7)
**> 50 cases**	371/554 (67.0)	379/556 (68.2)	361 (67.6)	370 (68.6)	365/527 (69.3)	373/546 (68.3)

© 2025 Rinne JKA et al. *JAMA Surgery*.

eTable 2 Operative details ITT

	ICG FI	Control group	p-value
**Intraoperative complication**	25/544 (4.6)	32/548 (5.8)	0.356
**Blood loss ml, mean (SD)**	84 (126)	84 (145)	0.944
**Change of anastomotic site No. (%)**	46/543 (8.5)	29/540 (5.4)	0.044*
**Conversions**	33/568 (5.8)	27/569 (4.8)	0.763
**Protective stoma**	11/546 (2.0)	12/551 (2.2)	0.850
**Operation**			0.176
**Right-sided No. (%)**	273/568) (48.1)	298/569 (52.4)	
**Left-sided No. (%)**	267/568 (47.0)	243/569 (42.7)	
**Other (colectomies, transversum resections)**	28/568 (5.0)	28/569 (4.9)	
**Operation time, mins, mean (SD)**	163 (64)	157 (64)	0.076
**Overall anastomotic leakage No. (%)**	33/567 (5.8)	45/567 (7.9)	0.159
**Right-sided No. (%)**	16/273 (5.9)	20/298 (6.7)	
**Left-sided No. (%)**	14/267 (5.2)	23/243 (9.5)	
**Postoperative infectious complications No. (%)**			0.678
**Antibiotics only**	2/383 (0.5)	3/391 (0.7)	
**Percutaneous drainage**	3/383 (0.7)	3/391 (0.8)	
**Reoperation No. (%)**	31/383 (8.1)	41/391 (10.5)	
**Overall reoperations No. (%)**	53/555 (9.5)	55/556 (9.9)	0.847
**Overall complications**	182/543 (33.5)	197/542 (36.3)	0.328
**Clavien-Dindo classification No. (%)**			0.499
**I**	31/447 (6.9)	43/460 (9.3)	
**II**	116/447 (26.0)	112/460 (24.3)	
**III**	54/447 (12.1)	58/460 (12.6)	
**IV**	7/447 (1.6)	6/460 (1.3)	
**V**	3/447 (0.7)	8/460 (1.7)	
**Postoperative hospital stay, days (SD)**	5.6 (5)	5.6 (5)	0.97

© 2025 Rinne JKA et al. *JAMA Surgery*.

eTable 3 Comparison of anastomoses

	mITT	As Treated
**Right side**		
**Intervention Group, intracorporeal endostapler**	162/294 (55.1 %)	142/271 (52.4 %)
**Control Group, intracorporeal endostapler**	146/274 (53.3 %)	166/297 (55.9 %)
**Intervention Group, extracorporeal linear stapler**	116/294 (39.5 %)	117/271 (43.2 %)
**Control Group, extracorporeal linear stapler**	114/274 (41.6 %)	113/297 (38 %)
**Intervention Group, hand-sewn**	15/294 (5.1 %)	11/271 (4.1 %)
**Control Group, hand-sewn**	13/274 (4.7 %)	17/297 (5.7 %)
**Intervention Group, circular stapler**	1/294 (0.3 %)	1/271 (0.4 %)
**Control Group, circular stapler**	1/274 (0.4 %)	1/297 (0.3 %)
**Left side**		
**Intervention Group, intracorporeal endostapler**	12/250 (4.8 %)	8/245 (3.3 %)
**Control Group, intracorporeal endostapler**	9/232 (3.9 %)	13/237 (5.5 %)
**Intervention Group, extracorporeal linear stapler**	13/250 (5.2 %)	10/245 (4.1 %)
**Control Group, extracorporeal linear stapler**	21/232 (9.1 %)	24/237 (10.1 %)
**Intervention Group, hand-sewn**	14/250 (5.6 %)	14/245 (5.7 %)
**Control Group, hand-sewn**	12/232 (5.2 %)	12/237 (5.1 %)
**Intervention Group, circular stapler**	211/250 (84.4 %)	213/245 (86.9 %)
**Control Group, circular stapler**	190/232 (81.9 %)	188/237 (79.3 %)
**Transverse colon**		
**Intervention Group, intracorporeal endostapler**	1/1 (100 %)	1/1 (100 %)
**Control Group, intracorporeal endostapler**	1/6 (16.7 %)	1/6 (16.7 %)
**Intervention Group, extracorporeal linear stapler**	0	0
**Control Group, extracorporeal linear stapler**	2/6 (33.3 %)	2/6 (28.6 %)
**Intervention Group, hand-sewn**	0	0
**Control Group, hand-sewn**	3/6 (50.0 %)	3/6 (50 %
**Intervention Group, circular stapler**	0	0
**Control Group, circular stapler**	0	0
**Other location**		
**Intervention Group, intracorporeal endostapler**	1/11 (9.1 %)	1/11 (9.1 %)
**Control Group, intracorporeal endostapler**	3/9 (33.3 %)	3/9 (33.3 %)
**Intervention Group, extracorporeal linear stapler**	5/11 (45.4 %)	4/11 (36.4 %)
**Control Group, extracorporeal linear stapler**	1/9 (11.1 %)	2/9 (22.2 %)
**Intervention Group, hand-sewn**	2/11 (18.2 %)	3/11 (27.3 %)
**Control Group, hand-sewn**	4/9 (44.4 %)	3/9 (33.3 %)
**Intervention Group, circular stapler**	3/11 (27.3 %)	3/11 (27.3 %)
**Control Group, circular stapler**	1/9 (11.1 %)	1/9 (11.1 %)

© 2025 Rinne JKA et al. *JAMA Surgery*.

eTable 4 ITT. Time to maximal signal intensity seconds, mean (SD)

	No leakages	With leakages	p-value
**Planned site**	47 (27)	50 (26)	0.629
**Proximal to anastomosis**	42 (17)	42 (19)	0.838
**Distal to anastomosis**	45 (18)	46 (19)	0.970
**Combined time**	128 (55)	132 (58)	0.697

© 2025 Rinne JKA et al. *JAMA Surgery*.

eTable 5 ITT. Intensity scores 0-2. No. (%)

	No leakages	With leakages	p-value
**Planned site**			0.57
**0**	7/464 (1.5)	0/30 (0)	
**1**	33/464 (7.1)	1/30 (3.3)	
**2**	424 (91.4)	29/30 (96.7)	
**Proximal to anastomosis**			0.958
**0**	0/459 (0)	0/29 (0)	
**1**	15/459 (3.3)	1/29 (3.4)	
**2**	444/459 (96.7)	28/29 (96.6)	
**Distal to anastomosis**			0.93
**0**	2/453 (0.4)	0/29 (0)	
**1**	34 (7.5)	2/29 (6.9)	
**2**	417 (92.1)	27/29 (93.1)	

© 2025 Rinne JKA et al. *JAMA Surgery*.

eTable 6 mITT. Time to maximal signal intensity, seconds, mean (SD)

	No leakages	With leakages	p-value
**Planned site**	47 (27)	50 (26)	0.634
**Proximal to anastomosis**	42 (17)	42 (19)	0.830
**Distal to anastomosis**	45 (18)	42 (19)	0.970
**Combined time**	133 (50)	132 (58)	0.938

© 2025 Rinne JKA et al. *JAMA Surgery*.

eTable 7 mITT. Intensity scores 0-2. No. (%)

	No leakages	With leakages	p-value
**Planned site**			0.556
**0**	7/454 (1.5)	0/30 (0)	
**1**	33/454 (7.3)	1/30 (3.3)	
**2**	414/454 (91.2)	29/30 (96.7)	
**Proximal to anastomosis**			0.975
**0**	0/449 (0)	0/29 (0)	
**1**	15 (3.3)	1/29 (3.4)	
**2**	434 (96.7)	28/29 (96.6)	
**Distal to anastomosis**			0.930
**0**	2/443 (0.5)	0/29 (0)	
**1**	33/443 (7.4)	2/29 (6.9)	
**2**	408/443 (92.1)	27/29 (93.1)	

© 2025 Rinne JKA et al. *JAMA Surgery*.

eTable 8 As Treated. Time to maximal signal intensity, seconds, mean (SD)

	No leakages	With leakages	p-value
**Planned site**	47 (27)	50 (26)	0.634
**Proximal to anastomosis**	42 (19)	42 (19)	0.830
**Distal to anastomosis**	46 (19)	46 (19)	0.970
**Combined time**	132 (58)	132 (58)	0.938

© 2025 Rinne JKA et al. *JAMA Surgery*.

eTable 9 As Treated. Intensity scores 0-2. No. (%)

	No leakages	With leakages	p-value
**Planned site**			0.556
**0**	7/454 (1.5)	0/30 (0)	
**1**	33/454 (7.3)	1/30 (3.3)	
**2**	414/454 (91.2)	29/30 (96.7)	
**Proximal to anastomosis**			0.975
**0**	0/449 (0)	0/29 (0)	
**1**	15/449 (3.3)	1/29 (3.4)	
**2**	434/449 (96.7)	28/29 (96.6)	
**Distal to anastomosis**			0.930
**0**	2/443	0/29	
**1**	33/443	2/29	
**2**	408/443	27/29	

© 2025 Rinne JKA et al. *JAMA Surgery*.

eTable 10 Comparison of Anastomotic leakages

	Anastomotic leakage ICG/Control No. (%)	OR	95% CI	p-value
**Intention to treat**	33/567 (5.8)<br>45/567 (7.9)	0.73	0.475-1.132	0.159
**Intention to treat**<br>**Right side**	16/273 (5.9)<br>20/298 (6.7)	0.87	0.462-1.650	
**Intention to treat**<br>**Left side**	14/267 (5.2)<br>23/243 (9.5)	0.55	0.292-1.052	
**Modified Intention to treat**	33/536 (6.2)<br>45/541 (8.3)	0.74	0.48-1.141	0.171
**Modified Intention to treat**<br>**Right side**	16/278 (5.8)<br>20/294 (6.8)	0.86	0.454-1.622	
**Modified Intention to treat**<br>**Left side**	14/250 (5.6)<br>23/232 (9.9)	0.57	0.298-1.071	
**As Treated**	31/528 (5.9)<br>47/549 (8.6)	0.686	0.443-1.062	0.089
**As Treated**<br>**Right Side**	15/271 (5.5)<br>21/279 (7.1)	0.78	0.412-1.487	
**As Treated**<br>**Left Side**	13/245 (5.3)<br>24/237 (10.1)	0.52	0.273-1.005	

© 2025 Rinne JKA et al. *JAMA Surgery*.
