# Supplementary material for: Indocyanine Green Fluorescence Imaging in Prevention of Colorectal Anastomotic Leakage: A Randomized Clinical Trial
Source: JAMA Surg. 2025 Mar 5;160(5):486–93. doi: 10.1001/jamasurg.2025.0006 (PMC11883591; doi:10.1001/jamasurg.2025.0006)
Supplement: Supplement 3. — Data sharing statement [file jamasurg-e250006-s003.pdf]

Data Sharing Statement

Rinne. Indocyanine Green Fluorescence Imaging in Prevention of Colorectal Anastomotic Leakage. *JAMA Surg*. Published May 14, 2025. doi:10.1001/jamasurg.2025.0006

Data
Additional Information: The study was registered on ClinicalTrials.gov (identifier NCT03602677), https://clinicaltrials.gov/study/NCT03602677.
Data available: No

Additional Information
Explanation for why data not available: Finnish laws for patient confidality are extremely strict
